# Assessing the Influence of Banana Leaf Ash as Pozzolanic Material for the Production of Green Concrete: A Mechanical and Microstructural Evaluation

**DOI:** 10.3390/ma17030720

**Published:** 2024-02-02

**Authors:** Md. Hamidul Islam, David William Law, Chamila Gunasekara, Md. Habibur Rahman Sobuz, Md. Nafiur Rahman, Md. Ahsan Habib, Ashanul Kabir Sabbir

**Affiliations:** 1 Department of Civil and Infrastructure Engineering, RMIT University, Melbourne, VIC 3000, Australia; s3835484@student.rmit.edu.au (M.H.I.); chamila.gunasekara@rmit.edu.au (C.G.); 2 Department of Building Engineering and Construction Management, Khulna University of Engineering & Technology, Khulna 9203, Bangladesh; habib@becm.kuet.ac.bd (M.H.R.S.); nafiurrahmanpial@gmail.com (M.N.R.); ahsanhabib570@gmail.com (M.A.H.); aksabbir14@gmail.com (A.K.S.)

**Keywords:** pozzolanic material, banana leaf ash, green concrete, mechanical properties, non-destructive testing (NDT), microscopic properties

## Abstract

This paper reports an investigation of the mechanical and microscopic properties of partially replaced banana leaf ash (BLA) concrete. In this research, the cement was partially replaced by BLA in two phases: Phase A (0%, 5%, 10%, 15%, 20%, 25% and 30%) and Phase B (0%, 10%, 20% and 30%). The consequence of partially replacing cement with BLA in concrete was investigated by the application of a range of tests, namely X-ray fluorescence (XRF), compressive strength, splitting tensile strength, flexure strength, ultrasonic pulse velocity and scanning electron microscopy (SEM) analysis. The properties were then correlated with the properties of a standard 100% Portland cement concrete of similar strength. The XRF result of the BLA identified a composition with 48.93% SiO_2_ and 3.48% Al_2_O_3,_ which indicates that the material potentially possesses pozzolanic properties. The mechanical properties of the partially replaced BLA concrete noted minor strength loss, approximately 5% with 20% partial replacement. The nondestructive testing data showed enhanced performance up to 20% partial replacement, with ultrasonic pulse values above 3500 m/s. The scanning electron microscopy analysis illustrated that the morphology of BLA specimens contained increased microcracks compared with the control. The decrease in strength observed is attributed to the fibrous composition of the BLA. The mechanical, nondestructive testing and microscopic results highlight the potential to utilize BLA as a partial replacement for cement as a pozzolanic material in concrete at up to 20% by weight of cement.

## 1. Introduction

Concrete is a vital and widely used construction material due to its strength, durability and availability of constituents across the globe. Day by day, the increasing usage of conventional concrete in the construction industry is causing the depletion of natural resources in the extraction process, which leads to damage to the landscape, the contamination of water and reduced air quality [1]. Moreover, though cement is an effective binder, the cost of production is significantly higher and generates significant quantities of CO_2_, which has far-reaching impacts on the environment [2,3]. The production of each ton of cement produces a total of 0.65~0.94 tons of CO_2_, where 55% is generated from the breakdown of calcium carbonate into calcium oxide (lime) and an additional 39% comes from fuel emissions [4,5,6]. The exact quantities depend on the modernity of the technology in the cement plant and the ratio of clinker to cement [7]. The “ratio of clinker to cement” refers to the proportion of clinker, a key component in the production of cement, relative to the total amount of cement in a mixture. It has been estimated that worldwide CO_2_ emission from the cement industry will be approximately 2.34 Gt/year in the year 2050 [8]. In addition, cement production requires a significant amount of energy, about 7.36 × 10^6^ KJ per ton of cement [3,9]. Therefore, the production of green concrete is necessary to preserve the environment. Green concrete is an environmentally friendly construction material that incorporates recycled or alternative components, reducing its carbon footprint and promoting sustainability throughout its production and life cycle. Many researchers have identified the utilization of waste materials and industrial bio-products as an appropriate option to produce green concrete. These studies include the use of crumb rubber [10], waste glass as an aggregate in concrete [11] and various other by-products such as rice husk ash [12], fly ash [13], bamboo leaf ash [14], corn cob [15], olive [16], sisal [17] and slag [18].

These materials were selected for use as partial replacements for locally available Portland cement (PC) due to their high silica and/or alumina content. Normally, during the pozzolanic reaction, hydrated phases are formed which enhance the performance of concrete [19,20,21,22,23]. In addition, these materials have the potential to reduce the costs of production and decrease environmental impacts, while maintaining a satisfactory mechanical and durability performance. Hence, they can fulfill the role of supplementary cementitious materials (SCMs) in concrete, which are technically realistic, sustainable and effective [24,25,26,27]. The partial incorporation of SCM in cement is a potentially beneficial method to reduce environmental impact and depletion [28]. In natural microfiber ash that is partially incorporated in cement, the fiber not only acts as cementitious material but can also lower aggregate quantities. Furthermore, natural fiber ash has been reported to increase compressive strength to a greater extent compared with other fibers such as polymer, glass and steel [27].

In recent years, the use of mineral admixtures in mortar and concrete has been broadly illustrated to increase the strength and durability of the composites, decrease costs and reduce cement consumption with substitution rates of approximately 8–10% [24,25,29,30,31,32,33,34,35]. The combustion of agricultural solid waste for the addition of the ash to concrete is a common practice due to the chemical reaction of the ash with the portlandite which is generated during cement hydration. Agricultural wastes, such as rice husk ash (RHA), sawdust, cork granules, coconut pith, sugar cane bagasse ash, wheat straw ash and sugar cane straw ash, have been proposed as SCMs for concrete production [36,37,38,39,40]. According to ASTM C125-13 [41], a pozzolan material is defined as a product that has a chemical structure containing siliceous or siliceous and aluminous constituents and minimal or no cement, but which will, in a finely divided form in water, react chemically with calcium hydroxide at ambient temperatures to form complex compounds that have cementitious characteristics. The pozzolanic reactivity arises from the reaction of amorphous silica with Ca(OH)_2_ to form calcium silicate hydrate gel, as represented in Equation (1).

xSiO_2_ + yCaO + zH_2_O ↔ xCaO · ySiO_2_ · zH_2_O (1)

In 2012, more than 95 million tons of banana and plantain was produced in Bangladesh [42,43]. Around 10.22 million tons of residue and ashes is produced worldwide from banana plants [26]. Banana fiber is a common agricultural waste in Bangladesh. Approximately 1 million tons of bananas is produced in Bangladesh per annum [44]. The interest in green technology and eco-friendly materials is increasing day by day, as such, the interest in and utilization of banana fiber is increasing in many diverse fields [45,46]. These include drilling cables, manufacturing of shipping cables, power transmission robs and cordage, apparel garments, home furnishings, etc. [45,46,47].

Banana leaf ash (BLA) can be classified as a pozzolanic material for the partial replacement of cement based on its chemical composition, containing 48.7% SiO_2_ and 2.6% Al_2_O_3,_ satisfying the requirements of a pozzolan [48]. Previous studies [6,26,32,49] have examined BLA at up to 10% replacement as a potential pozzolanic material for use in cement mortars and regular concrete.

Researchers worldwide are actively exploring the use of waste materials to develop more environmentally friendly products. Numerous waste products, including waste ceramic powders, palm oil fuel ash, synthetic fibers, fly ash, foundry sand waste, crumb rubber, coconut shell aggregate, recycled aggregates, polypropylene fibers, rice husk ash and others, have been utilized in concrete to partially replace coarse aggregates, fine aggregates and cement. However, very few researchers have utilized BLA as an SCM in concrete. Since a banana tree bears fruit only once, the tree becomes redundant after fruit harvesting. Consequently, BLA can be a cost-effective substitute for cement while also contributing to a reduction in global warming.

As noted, to date, scant research has investigated cases where more than 10% of cement was replaced by BLA, with little data on the mechanical and microscopic properties of partially replaced BLA concrete being reported. There is insufficient data available on the mechanical and microscopic properties of concrete that incorporate partially replaced BLA concrete to enable its application in reinforced areas with close spacing, as well as in structures that are resistant to segregation. Moreover, incorporating BLA in concrete presents a more environmentally friendly and cost-effective alternative [49]. Additionally, the characteristics of a banana tree also help to make it cost-effective [5,46,49]. A banana tree can produce fruit only once in its life cycle, and after harvesting the fruit, the fiber is directly dumped into landfills or decomposed as solid organic waste. Hence, BLA could be used as a partial replacement for cement almost free of cost.

The potential utilization of BLA as an SCM due to its potential pozzolanic properties suggested by its chemical composition is considerable. This study reports its concrete properties by incorporating varying concentrations of BLA as a partial replacement for the binder. The investigation primarily focuses on examining the engineering characteristics to produce sustainable concrete. In this study, various samples were prepared to replace cement with BLA in two phases: Phase A (0%, 5%, 10%, 15%, 20%, 25% and 30%) by weight of binder and Phase B (0%, 10%, 20% and 30%). In Phase A, mechanical and nondestructive testing (NDT) was conducted. On the basis of these results, Phase B assessed the flexural and microscopic properties of the BLA concrete. The main purpose of this study is to investigate the effects of BLA as a pozzolan to enhance green concrete development.

## 2. Materials and Methods

### 2.1. Production and Characterization of Ash

The production of BLA involved subjecting it to combustion at a relatively high temperature, specifically at approximately 650 °C for a period of 75 min. The burning process was conducted following the recommended guidelines, with a controlled burning rate of 10 °C per minute [5,6,19,26,30,50,51,52]. To maximize the amorphous content, the temperature and burning time were kept constant with those applied for BLA powders [26]. Following burning, the material was grounded in a ball mill with a capacity of 35 L at 55 rpm for 30 min. The BLA was sieved through #200, and particles size <75 µm were utilized for the replacement of cement. Analysis of the XRD data revealed that approximately 84.0% of the ash consisted of an amorphous phase, while the remaining crystalline phases were identified as calcite (CaCO_3_), quartz (SiO_2_) and magnesium carbonate (MgCO_3_) [49]. The presence of these crystalline forms can be attributed to the gradual cooling of the BLA inside the oven, as the ash maintained temperatures of around 650 °C for a duration of 8 h until it was removed from the oven. 

**Figure 1:**
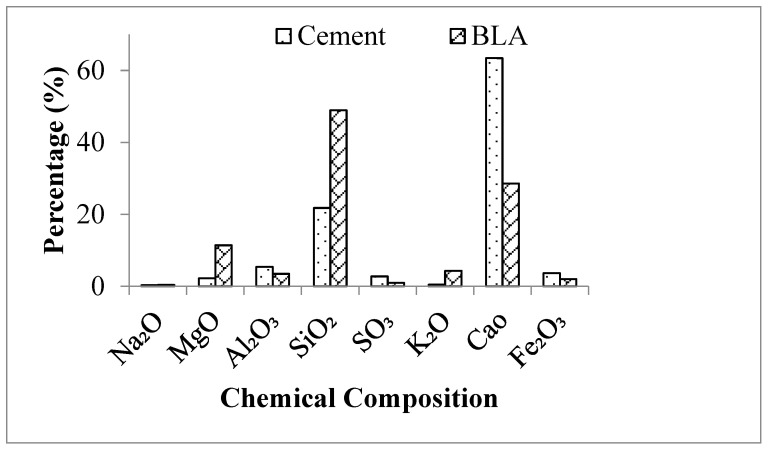
Chemical composition cement and BLA.

The chemical composition of BLA was analyzed through X-ray fluorescence (XRF) using a Bruker Axs S4 Pioneer, USA, and the results obtained are reported in Figure 1. The oxides of BLA, Na₂O, MgO, SO₃, K₂O, Cao and Fe₂O₃ showed satisfactory percentages in comparison with cement. Silicon dioxide (SiO_2_) is a vital chemical component in pozzolanic materials as, at an ambient temperature, it will react with calcium hydroxide to form compounds with cementitious characteristics. Through this reaction, calcium silicate hydrate gel is produced, which is expected to fill the voids and enhance the strength of concrete. In this research, BLA is anticipated to act as a pozzolanic material due to its chemical composition, as derived from the XRF analysis, containing 48.93% SiO_2_ and 3.48% Al_2_O_3,_ which indicates a potentially pozzolanic character.

To produce mortar and concrete samples, ordinary Portland cement according to ASTM C150 Type I [53] was used together with natural sand and granite aggregates. The specific gravity, water absorption, fineness modulus and unit weight of the aggregate were determined according to ASTM C127 [54], ASTM C128 [55], ASTM C136 [56] and ASTM C29 [57] standards, respectively. Table 1 shows the gradation and physical properties of the aggregates. A mix design based on a 95% confidence interval, with characteristic strength *f_c_* = 25 MPa and a target mean strength *f_m_* = 30 MPa, was prepared following the Absolute Volume Method ACI 211. Seven and five mixes (Phase A and Phase B) with the replacement of cement by BLA by weight % of cement were prepared, as shown in Table 2. The specimens’ code relates to the percentage of BLA by weight of the replacement of cement, i.e., 0% meaning that the specimen is made with 100% cement and “0 wt.%–BLAz” as a binder and 10% meaning the specimen is made with 90% cement and “10 wt.%–BLA” as a binder.

In this research, the equivalent BLA concrete and/or mortar specimens for each mix were obtained by replacing the cement with the designated percentage by weight of BLA; all other constituents remained the same. Table 3 and Table 4 show the gradation properties of the fine and coarse aggregates, respectively.

To ensure a dry condition of the saturated surface, the granite aggregates were soaked in water for 24 h and then air-dried before mixing with other elements. A drum mixer was used for mixing, and the workability of the fresh concrete was determined by a standard slump cone test. The test samples were cast in steel molds and compacted by stroke rod as per ASTM C143 standards [58]. Concrete and mortar samples were demolded 1 day after casting.

### 2.2. Concrete Mix—Design and Characterization

#### 2.2.1. Compressive Strength of Cubic Mortar Specimens

The compressive strength of cubic mortar specimens was determined by following ASTM C109 [59] using a Controls automatic compression tester, Italy. The size of the cubic specimens was 50 mm × 50 mm × 50 mm. The mixed proportions for the investigations were 1:2.75 for cement: sand and 1:0.485 for water: binder. The specimens were cured at a temperature equal to 19 ± 1 °C in water until reaching the testing age. Compressive strength was determined from an average of three cubic mortar samples tested after 7 and 28 days of curing.

#### 2.2.2. Compressive Strength of Cylindrical Concrete Specimen

The compressive strength of the cylindrical concrete specimens was determined according to ASTM C39 [60] using a Controls automatic compression tester, Italy. The test was conducted on 100 mm diameter × 200 mm height cylindrical specimens with mix proportions 1.0:1.5:3.0 (cement: sand: aggregate) and 0.5:1.0 (water:binder) maintaining a slump value of 100 ± 5 mm. The specimens were cured at a temperature equal to 19 ± 1 °C and stored underwater until testing. Compressive strength was determined from an average of three cylindrical samples tested at the ages of 7 and 28 days of curing.

#### 2.2.3. Splitting Tensile Strength of Cylindrical Concrete Specimen

The splitting tensile strength samples preparation, mix ratios and curing conditions were the same as the compressive strength test of cylindrical concrete specimens. Splitting tensile strength was obtained according to ASTM C496 [61] using a Controls split tensile tester, Italy.

#### 2.2.4. Flexural Strength of the Concrete Beam

**Figure 2:**
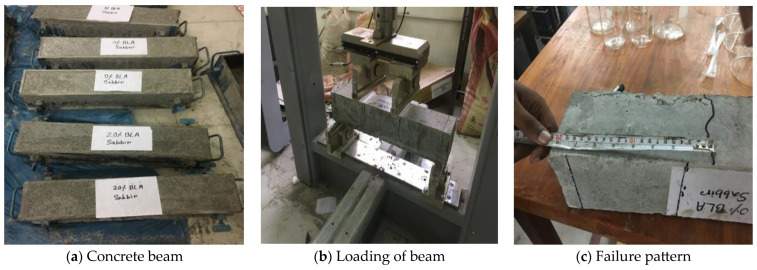
Flexural strength test of concrete beam.

The flexural strength test of the concrete beam was conducted according to ASTM D6272 [62] using a Controls automatic flexure tester, Italy, Figure 2. To perform the flexural strength test, three concrete beams (100 mm × 100 mm × 500 mm) were prepared. The mix ratio, w/c ratio and curing conditions were the same as the compressive strength test of concrete. Flexure strength was determined after 7 and 28 days of curing. A four-point loading procedure was used. The load was applied continuously at a rate of movement corresponding to a rate of increase in stress on the sample of 1.50 ± 0.25 MPa per minute until rupture occurred. 

#### 2.2.5. Scanning Electron Microscopy Analysis

The scanning electron microscopy (SEM) analysis was conducted by an EVO 18 Research SEM machine, Germany. The samples were dry cut into 10 mm^3^ cubes using a diamond saw cutter, covered in epoxy resin and polished. Once completed, samples were placed in a sample holder, secured by carbon tape and connected to the sample holder by electric or aluminum tape. The function of carbon tape was to hold the sample in place. The function of the electric or aluminum tape was to provide an electrically conductive connection between the sample surface and sample holder. The group sample holder was then placed in a SPUTTER Coater, Germany. The machine used the gold conductive process. A group sample holder was placed in the SEM machine for testing. 

#### 2.2.6. Ultrasonic Pulse Velocity Test

An ultrasonic pulse velocity (UPV) test was conducted on concrete to indicate the homogeneity of concrete, as well as the presence of voids, cracks, and other imperfections. UPV is a nondestructive testing method that assesses the quality of concrete. In this study, the transmission time was also obtained. The UPV test was conducted on cylindrical concrete specimens of size 100 mm diameter × 200 mm height at 28 days of curing.

The cylindrical concrete specimen was removed from the curing tank and then dried in the sun for 48 h. The average room temperature was 26 °C and average relative humidity was 65.8%. Two probes (transducer and receiver) were applied at the top and bottom faces of the specimen in a noiseless controlled environment. The pulse velocity and transmission time were measured. 

## 3. Results

### 3.1. BLA as Partial Replacement of Cement (0%, 5%, 10%, 15%, 20%, 25% and 30%)—Phase A

#### 3.1.1. Compressive Strength of Mortar Specimens

In Table 5, a summary of the compressive strength test results is provided, including the mean strength, coefficient of variation, standard deviation and standard error, as well as the lower and upper bounds of the 95% confidence interval. The findings indicate a gradual decline in compressive strength over time with an increase in BLA %. The 7-day strength ranged from 25.28 MPa (BLA0) to 20.80 MPa (BLA30), while for the 28-day testing period, it ranged from 32.88 MPa (BLA0) to 26.96 MPa (BLA30). The standard deviation for the 7-day tests varied between 0.325 and 0.114, with corresponding standard errors of approximately 0.188 to 0.066. The coefficient of variation for the same period ranged from 1.3% to 0.5%. Similarly, for the 28-day tests, the standard deviation ranged between 0.280 and 0.101, with standard errors of 0.162 to 0.058. The coefficient of variation fluctuated between 0.9% and 0.3%. The coefficients of variation are relatively small [63]. This is attributed to the homogeneity of the mix coupled with the specimens’ size and compaction applied.

The control mix, BLA0, had the highest compressive strength: 25.28 MPa after 7 days. Conversely, the BLA30 mixture exhibited the minimum compressive strength: 20.80 MPa. The compressive strengths of the remaining four mixtures, specifically BLA10, BLA15, BLA20 and BLA25, were 24.82 MPa, 23.93 MPa, 23.04 MPa and 22.72 MPa, respectively. The compressive strength of the specimens attained a maximum of 32.88 MPa at 28 days and a minimum of 26.96 MPa. The results demonstrate a significant variance in compressive strength at 7 days, but this variation reduced considerably after 28 days. 

**Figure 3:**
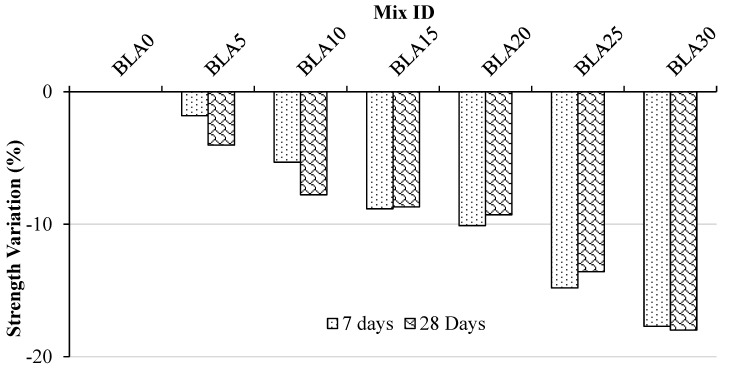
Variation in compressive strength of mortars at 7 and 28 days as compared with the control (BLA0).

Figure 3 depicts the relationship between compressive strength and the BLA specimens in comparison with the control mixture. After 7 and 28 days, the concrete mix containing 30% BLA exhibited a reduction in strength of approximately 17.71% and 18.00%, respectively. For concrete mixes with 10%, 15%, 20% and 25% BLA, the compressive strengths measured were 24.82 MPa, 23.93 MPa, 23.04 MPa, 22.72 MPa and 21.53 MPa. These values were lower than the control mix by 1.81%, 5.31%, 8.84%, 10.10% and 14.81% after 7 days. Similarly, at 28 days, the compressive strengths were 31.55 MPa, 30.32 MPa, 30.02 MPa, 29.82 MPa and 28.41 MPa, which are lower than the control mix by 4.03%, 7.78%, 8.69%, 9.29% and 13.59%.

There is a noticeable difference in compressive strength variations between 7 days and 28 days for different BLA mixes. The 20% and 25% BLA mixes show lower variations in compressive strength at 28 days compared with 7 days, whereas the 5% and 10% mixes exhibit slightly higher variations. On the other hand, the 15% and 30% BLA mixes display similar variations in compressive strength at both 7 and 28 days. These results suggest that when cement is partially replaced with BLA, the compressive strengths increase with increased curing duration. This improvement can be attributed to the effective water absorption and filling of porous spaces within the concrete mass by the BLA mix. Consequently, the compressive strength is enhanced with extended curing. Specifically, for the 20% BLA mix, the strength reduction is only around 9% at 28 days compared with the control mix, and it is lower than the 7-day curing period. These findings indicate that a 20% BLA replacement is the optimal choice for maintaining compressive strength and maximizing BLA percentage replacement.

#### 3.1.2. Compressive Strength of Cylindrical Concrete Specimens

The compressive strength test results are summarized in Table 6, including mean strength, coefficient of variation, standard deviation, standard error and the lower and upper bounds of the 95% confidence interval. The findings indicate that as the amount of BLA percentage used as a cement substitute increases, the compressive strength gradually decreases. Specifically, the strength ranged from 28.29 MPa to 22.57 MPa at 7 days and from 36.31 MPa to 29.02 MPa at 28 days. For the 7-day period, the standard deviation varied from 0.375 to 0.111, the standard error ranged from 0.217 to 0.064 and the coefficient of variation was approximately 1.66% to 0.44%. Similarly, at 28 days, the standard deviation ranged from 0.438 to 0.112, the standard error varied from 0.253 to 0.065 and the coefficient of variation was approximately 1.43% to 0.32%.

At the 7-day mark, the BLA30 mix (30% BLA replacement) showed the lowest compressive strength of approximately 22.57 MPa, with 95% confidence intervals ranging from 21.638 MPa to 23.502 MPa, while the BLA0 (control mix) exhibited the highest strength of around 28.29 MPa, with 95% confidence intervals ranging from 27.80 MPa to 28.78 MPa. Similarly, at 28 days, the BLA30 mix displayed the lowest compressive strength of about 29.02 MPa, with 95% confidence intervals ranging from 28.72 MPa to 29.32 MPa, while the BLA0 mix gave the highest strength of approximately 36.31 MPa, with 95% confidence intervals ranging from 35.91 MPa to 36.72 MPa. The substitution of cement, the primary binding ingredient, with a less reactive material, BLA, resulted in a decrease in strength.

**Figure 4:**
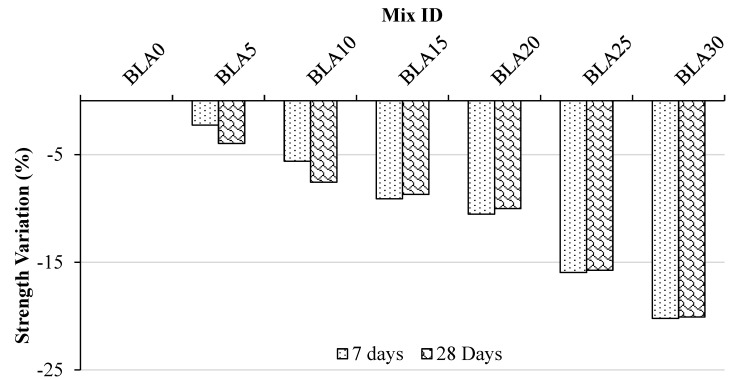
Variation in compressive strength of concretes at 7 and 28 days as compared with the control (BLA0).

Figure 4 illustrates the relationship between compressive strength and varying concentrations of BLA replacement compared with the control mixture. After 7 and 28 days, the concrete mix with 30% BLA experienced reductions in strength of approximately 20.21% and 20.08%, respectively. Concrete mixes containing 5%, 10%, 15%, 20% and 25% BLA had compressive strengths of 27.65 MPa, 26.70 MPa, 25.71 MPa, 25.31 MPa and 23.77 MPa after 7 days, representing decreases of 2.25%, 5.62%, 9.10%, 10.52% and 15.96% compared with the control mix. Similarly, at 28 days, the compressive strengths were 34.88 MPa, 33.57 MPa, 33.15 MPa, 32.68 MPa and 30.59 MPa, reflecting reductions of 3.96%, 7.55%, 8.70%, 10.01% and 15.75% compared with the control mix. The increase in strength from 7 to 28 days was less prominent in the concrete specimens than in the mortar specimens. Notably, replacement levels of 20% and above showed similar strength reductions compared with the control at both 7 and 28 days. The compressive strength of the concrete, up to a 20% partial replacement of cement by BLA, was approximately 25.31 MPa and 35.68 MPa at 7 and 28 days in comparison with the control mix, as described in Table 6.

Previous research studies conducted by Vignesh et al. (2021), Pawar and Aman (2018) and Ndubuisi (2016) [64,65,66] support the observations of this study. These studies reported similar findings regarding varying concentrations of BLA replacement. Up to a 20% substitution of cement with BLA, a slight reduction in strength compared with the control mix was observed. However, beyond this threshold, the decrease in strength increases rapidly. This phenomenon can be explained by the fact that cement hydrates to provide the requisite binding capacity to achieve the specified strength in the 100% cement mortar and concrete mixes. Nevertheless, there are still some pores and voids present in the mortar or concrete matrix. As the BLA replacement increases and the cement decreases, the rate of hydration decreases due to the slower hydration of the BLA due to its pozzolanic nature. However, as the curing time extends, it is hypothesized that the BLA hydrates and fills these voids; thus, the strength is only slightly reduced compared with the 100% cement control. 

Extended curing enhances compressive strength, particularly for the 20% BLA mix, which exhibits only a 10.01% reduction at 28 days compared with the control mix. Based on this small reduction in strength, 20% BLA replacement is identified as the optimal option for achieving acceptable compressive strength while maximizing BLA content. 

#### 3.1.3. Splitting Tensile Strength of Cylindrical Concrete Specimen

Table 7 presents a summary of the findings from the split tensile strength tests. It includes the average strength, coefficient of variation, standard deviation, standard error, and the lower and upper bounds of the 95% confidence interval. The results indicate that the splitting tensile strength ranged from 1.92 MPa to 2.84 MPa after 7 days and from 3.08 MPa to 4.15 MPa after 28 days. In terms of variability, for the 7-day samples, the standard deviation varied from 0.036 to 0.101, with a standard error ranging from 0.021 to 0.059 and a coefficient of variation between 1.54% and 3.57%. Conversely, for the 28-day samples, the standard deviation ranged from 0.191 to 0.060, with a standard error of approximately 0.035 to 0.110 and a coefficient of variation between 1.85% and 5.19%.

The tensile strength of the control mixture, BLA0, was found to be 2.84 MPa and 4.15 MPa after 7 and 28 days, respectively. The findings indicate that the addition of BLA to the concrete resulted in a decrease in tensile strength compared with the control mix. For the 7-day period, the BLA30 mix with 30% replacement of BLA exhibited the lowest flexural strength at approximately 1.92 MPa. The 95% confidence intervals for this mix ranged from 1.76 MPa to 2.08 MPa. On the other hand, the control mix (BLA0) demonstrated the highest strength at 2.84 MPa, with 95% confidence intervals spanning from 2.59 MPa to 3.09 MPa. Similarly, for the 28-day period, the BLA30 mix displayed the lowest flexural strength at about 3.08 MPa, with 95% confidence intervals ranging from 2.89 MPa to 3.26 MPa. In contrast, the control mix (BLA0) exhibited the highest strength at approximately 4.15 MPa, with 95% confidence intervals spanning from 3.69 MPa to 4.62 MPa.

**Figure 5:**
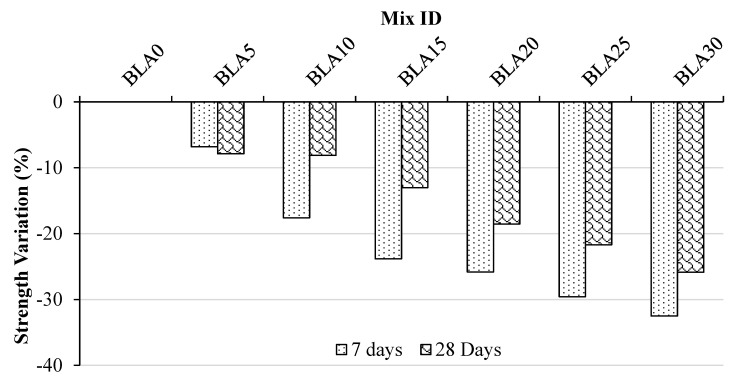
Variation in tensile strength of mortars at 7 and 28 days as compared with the control (BLA0).

Figure 5 illustrates the changes in split tensile strength as the concentration of BLA replacement varies in comparison with the control mixture. Over a period of 7 and 28 days, the concrete mixes with 5%, 10%, 15%, 20% and 25% BLA concentrations experienced reductions in strength of approximately 6.81%, 17.61%, 23.83%, 25.82% and 29.58%, respectively, while the 30% BLA displayed the highest impacts, with reductions of 32.51% after 7 days and 25.86% after 28 days.

Similar to compressive strength, the tensile strength showed a steady decrease in strength with a level of replacement up to 20% but a rapid decrease in strength for 25% and 30%, both at 7 days and 28 days. This would again indicate that a replacement level of 20% BLA is optimal for maintaining strength while maximizing replacement content. However, the reductions in tensile strength are greater than those for compressive strength. Tensile strength is more dependent on the interfacial transition zone (ITZ) than compressive, which supports the hypothesis that BLA adversely influences early-age hydration PC and is hence less effective in contributing to the ITZ strength, but due to the pozzolanic nature of the BLA, the hydration products from the BLA fill the voids and hence enhance compressive strength over time. The acceptable split tensile strength is approximately 2~5 MPa, as per [67]. In this study, the split tensile strengths of all BLA concrete mixes are within this acceptable range. 

#### 3.1.4. Correlation between Compressive Strength and Split Tensile Strength

**Figure 6:**
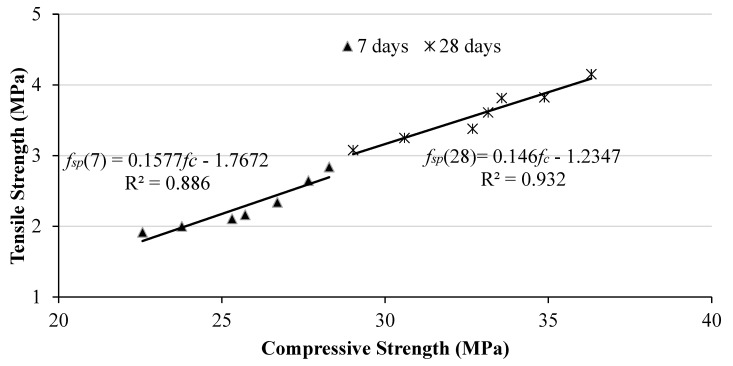
Correlation between compressive strength and splitting tensile strength.

In Figure 6, the correlation between compressive and splitting tensile strength of BLA concrete mixtures is depicted for both 7 and 28 days. The data exhibit a strong positive linear correlation, as indicated by the high determination coefficients of R^2^ = 0.886 and 0.932. The higher coefficients of determination demonstrate the good accuracy of the prediction with the experimental and analytical quantifications. It should be noted that this is a relatively small sample size and additional data would provide increased confidence in the predicted values. This mathematical correlation could be utilized to predict the hardened properties of concrete based on available data. By employing this prediction method, it may become possible to simulate critical components in a finite element analysis without the need for testing new materials of different ratios. This approach offers advantages such as the development of novel materials and mix proportions without incurring high costs, lengthy production times or the use of bulky equipment. The relationship between compressive and splitting tensile strength for all BLA % and control concrete mixtures at 7 and 28 days can be represented by Equations (2) and (3), where *f_sp_* represents the splitting tensile strength and *fc* represents the compressive strength.

*f_sp_*(7) = 0.1577*f_c_* − 1.7672 (2)

*f_sp_*(28) = 0.146*fc* − 1.2347 (3)

#### 3.1.5. Modulus of Elasticity of Concrete (*E_c_*)

*E_c_* can be calculated by the formula given by the ACI code:

(4) $$E_{c}=33{w_{c}}^{1.5}\sqrt{f_{c}^{/}}\;{\mathrm{lb}/\mathrm{in}}^{2}\mathrm{in}\;\mathrm{USCS}\;\mathrm{units}$$

(5) $$E_{c}=0.043{w_{c}}^{1.5}\sqrt{f_{c}^{/}}\;\mathrm{Mpa}\;\mathrm{in}\;\mathrm{SI}\;\mathrm{units}$$

With normal weight, normal density concrete, these two relations can be simplified to

(6) $$E_{c}=57,000\sqrt{f_{c}^{/}}\;{\mathrm{lb}/\mathrm{in}}^{2}\mathrm{in}\;\mathrm{USCS}\;\mathrm{units}$$

(7) $$E_{c}=4700\sqrt{f_{c}^{/}}\;\mathrm{Mpa}\;\mathrm{in}\;\mathrm{SI}\;\mathrm{units}$$

where

$f_{c}^{\prime }=$ specifies the 28-day compressive strength of concrete (lb/in^2^ or MPa);
$w_{c}=$ unit weight of concrete (lb/ft^3^ or kg/m^3^).

In this study, the modulus of elasticity was calculated utilizing Equation (7).

**Figure 7:**
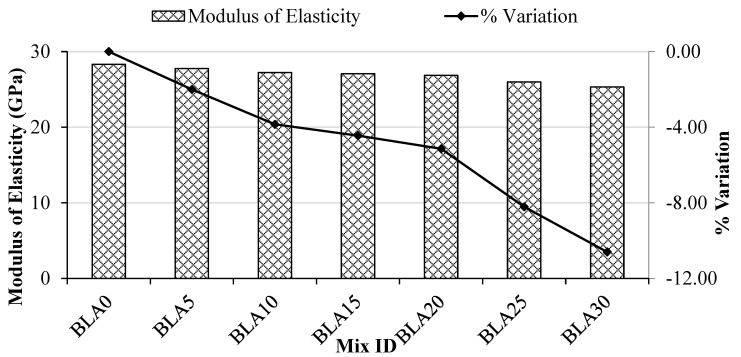
Modulus of elasticity compared with the control (BLA0).

The modulus of elasticity and the variation compared with the control mix at 28 days of curing age are illustrated in Figure 7. The modulus of elasticity of the control concrete mix was 28.32 GPa, and up to the 20% BLA mix, it maintained a range of 28 ± 2 GPa. The variation in the modulus of elasticity at up to 20% partial replacement by BLA demonstrated little reduction (only 5%) compared with the control mix, but at increased BLA content, this rose to more than 10%. This again demonstrates that the substitution of BLA to 20% maintains a suitable level of performance.

#### 3.1.6. NDT Results

**Figure 8:**
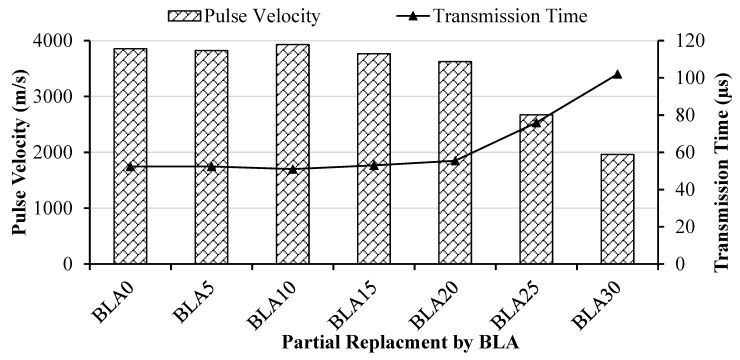
Pulse velocity and transmission time.

The pulse velocity and quality of concrete relationship is shown in Table 8. The ultrasonic pulse velocity and transmission time are given in Figure 8.

Pulse velocities and transmission times are almost linear up to 20% partial replacement, measuring approximately 3625 m/s and 50 µs, respectively. Beyond 20% BLA concrete mix, the pulse velocity decreases rapidly to 2670 m/s and transmission time increases, corresponding to medium-quality concrete. Although NDT is a qualitative test method, by comparing the pulse velocity and transmission with the standard values, it can be concluded that up to 20% replacement, the concrete can be classified as a good-quality concrete. 

### 3.2. BLA as Partial Replacement of Cement (0%, 10%, 20% and 30%)—Phase B

#### 3.2.1. Flexural Strength

Table 9 presents a summary of the findings from the flexural strength tests. It includes the average strength, coefficient of variation, standard deviation, standard error and the lower and upper bounds of the 95% confidence interval. The results indicate that the flexural strength varied from 3.71 MPa to 2.63 MPa after 7 days and from 5.27 MPa to 4.05 MPa after 28 days. In terms of variability, for the 7-day samples, the standard deviation ranged from 0.23 to 0.10, with a standard error ranging from 0.13 to 0.06 and a coefficient of variation between 8.23% and 3.63%. Conversely, for the 28-day samples, the standard deviation varied between 0.25 and 0.15, with a standard error ranging from 0.14 to 0.09 and a coefficient of variation between 4.92% and 3.13%.

The flexural strength of the control mixture, BLA0, was found to be 3.71 MPa and 5.27 MPa at 7 and 28 days, respectively. The results indicate that the addition of BLA to the concrete resulted in a decrease in its flexural strength compared with the control mix. For the 7-day period, the BLA30 mix, which involved a 30% replacement of BLA, exhibited the lowest flexural strength at approximately 2.63 MPa. The 95% confidence intervals for this mix ranged from 2.87 MPa to 2.39 MPa. On the other hand, the control mix (BLA0) demonstrated the highest strength at around 3.71 MPa, with 95% confidence intervals spanning from 4.17 MPa to 3.26 MPa. Similarly, for the 28-day period, the BLA30 mix displayed the lowest flexural strength at about 4.05 MPa, with 95% confidence intervals ranging from 4.42 MPa to 3.67 MPa. In contrast, the control mix (BLA0) exhibited the highest strength at approximately 5.27 MPa, with a 95% confidence interval spanning from 4.17 MPa to 3.26 MPa. The flexural strength data are in good agreement with the compressive and tensile strength data observed in Phase A.

**Figure 9:**
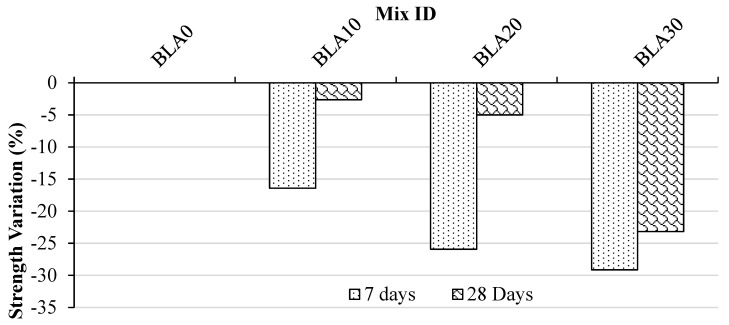
Variation in flexural strength of concrete mixes at 7 and 28 days compared with the control (BLA0).

Figure 9 illustrates the variation in flexural strengths. Over the periods of 7 and 28 days, concrete mixes with 10% and 20% BLA replacement exhibited decreases in strength compared with the control concrete. The flexural strengths increased as the curing period progressed, with minimal decreases observed up to a 20% partial cement replacement with BLA at 28 days of curing. The flexural strength of BLA20 was approximately 5.01 MPa, representing a 5% drop compared with the control mix. However, a significant decrease in strength was observed at the 30% BLA replacement. This is again attributed to the impact of the ITZ strength on the flexural strength development. The results again indicate that a 20% cement replacement content is optimal for BLA.

#### 3.2.2. Compressive Strength vs. Flexural Strength

**Figure 10:**
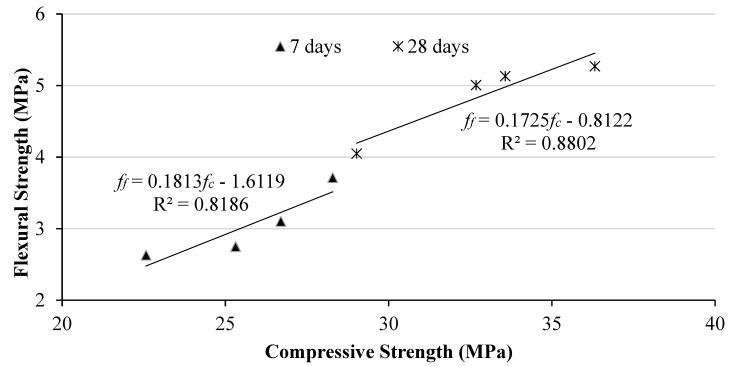
Correlation between compressive strength and flexural strength.

Figure 10 illustrates the correlation between compressive and flexural strength measurements. The data exhibit a strong positive linear correlation, as indicated by the high determination coefficients of R^2^ = 0.8186 and 0.8802. The high coefficients of determination indicate a strong correlation with both experimental and analytical quantifications. The relationship between compressive and flexural strength at 7 and 28 days can be represented by the following two equations, *f_f_ =* 0.1813*f_c_* − 1.6119 and *f_f_ =* 0.1725*fc* − 0.8122, respectively, where *f_f_* represents flexural strength and fc represents compressive strength.

#### 3.2.3. Microstructural Analysis

**Figure 11:**
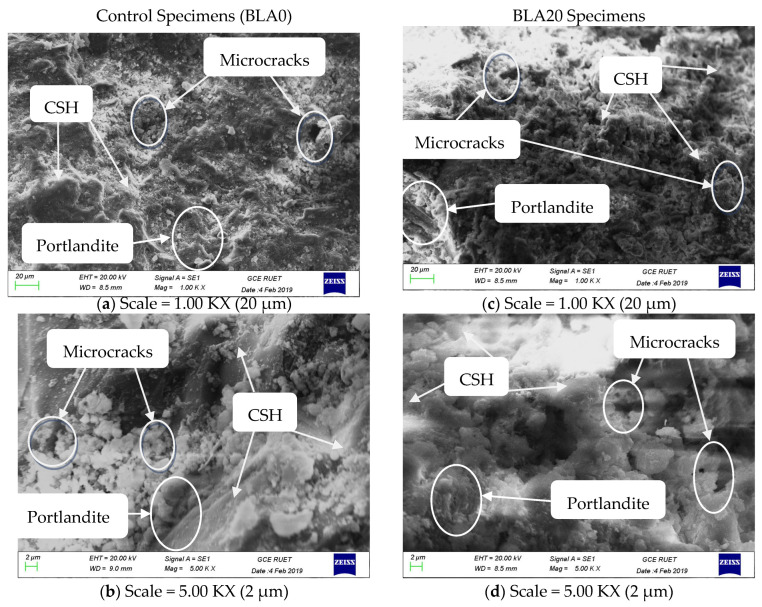
SEM analysis of control (BLA0) and 20% BLA (BLA20).

SEM analysis was conducted on the control and BLA20 concrete mix after 28 days of curing, as the chemical, mechanical and NDT results all indicate the 20% partially replaced BLA concrete mix provided the optimal balance between maintaining performance and maximizing cement replacement with BLA. The control concrete and the 20% BLA specimen at a resolution of 20 µm are shown in Figure 11. The typical appearance comprises particles of calcium oxide and hydration products Portlandite and C-S-H gel, as illustrated in the Figure 11. At the higher resolution of 2 µm, grains of partially reacted silica can be observed in Figure 11b,d. These are the shadow spheres that surround the light fibrous zone that comprises cementitious silica, an essential ingredient in concrete. 

Figure 11a,b demonstrates that the microstructural characteristics of the control specimen after 28 days of curing appear denser, with minimal quantities of calcium hydroxide and ettringite. However, some microcracks were evident in both specimens. Furthermore, Figure 11c,d reveals that the BLA specimens exhibit a net-like structural appearance, indicating the presence of secondary calcium silicate hydrate. A relative decrease in CSH and Portlandite was observed with increasing BLA content, which would account for the decrease in strength observed. The TGA analysis conducted by Mim et al., 2023 [49] supports this observation and notes that elevated temperatures were necessary to disrupt the configuration of the cementitious paste when BLA was incorporated. Hence, it is hypothesized that BLA10, BLA20 and BLA30 require more heat (energy) to evaporate water compared with control samples, which leads to an increase in microcracks observed in BLA specimens compared with the control. It was also noted that fewer microcracks were evident with the increase in BLA content. This may be due to the fibrous nature of BLA affecting the interfacial transition zone.

## 4. Conclusions

The feasibility and impact of using BLA as a partial substitute for cement were assessed by conducting mechanical, nondestructive testing (NDT) and microscopy testing. This study showed the following:

1. BLA has a chemical composition of 48.93% SiO_2_ and 3.48% Al_2_O_3,_ indicating that BLA has the potential to be a pozzolanic material.
2. Compressive, splitting tensile and flexure strength test results show that partial replacement with BLA of up to 20% resulted in minor strength loss (approximately 5%).
3. UPV analysis indicated that 20% partially replaced BLA concrete could be classed as good-quality concrete, the same rating as the control concrete.
4. SEM analysis revealed a lower quantity of microcracks in comparison with the control specimen. The slight decrease in strength observed is attributed to a weaker ITZ due to the fibrous nature of BLA, coupled with less CH and C-S-H being generated as the BLA content increases.
5. The splitting, tensile and flexural strengths were in the acceptable range, which is approximately 2~5 MPa, as per ACI-318. Tensile strength relies on the interfacial transition zone; BLA replacement inhibits early PC hydration but enhances later-age hydration, increasing compressive strength by filling voids. The data indicated a strong positive linear correlation between compressive and tensile or flexural strength.
6. The results indicate that BLA could be suitable as a partial replacement at up to 20% weight of cement, acting as a pozzolan to provide an alternative material in green concrete production.

Based on the initial results, a number of recommendations for future research are highlighted. Firstly, having demonstrated its potential as a pozzolanic material, additional testing should be undertaken to determine the pozzolanic activity of BLA. Secondly, additional data should be obtained to improve the reliability models derived to predict mechanical performance. 

## Figures and Tables

**Table 1:** Material properties.

Materials	Properties	Unit	Value
Fine Aggregate	Specific Gravity	-	2.65
	Absorption	%	2.12
	Fineness Modulus	-	2.87
	Unit Weight	Kg/m^3^	1495
Coarse Aggregate	Specific Gravity	-	2.55
	Absorption	%	1.35
	Fineness Modulus	-	7.06
	Unit Weight	Kg/m^3^	1570

**Table 2:** Mix proportions (kg/m^3^).

Mix ID	Cement (kg/m^3^)	BLA (kg/m^3^)	Sand (kg/m^3^)	Stone Chips (kg/m^3^)	Water (kg/m^3^)	Water/Binder Ratio
BLA0	300	0	450	900	150	0.50
BLA5	285	15	450	900	150	
BLA10	270	30	450	900	150	
BLA15	255	45	450	900	150	
BLA20	240	60	450	900	150	
BLA25	225	75	450	900	150	
BLA30	210	90	450	900	150	

**Table 3:** Gradation characteristics of fine aggregates.

Sieve Size	Sieve Opening (mm)	Weight Retained (gm)	Cumulative Weight Retained (gm)	Cumulative Weight Retained (%)	% Finer	Fineness Modulus Value
#4	4.750	0.00	0.00	0.00	100.00	2.55
#8	2.360	25.50	25.500	5.10	94.90	
#16	1.180	85.50	111.00	22.20	77.80	
#30	0.600	162.00	273.00	54.60	45.40	
#50	0.300	121.75	394.75	79.95	20.05	
#100	0.150	71.25	466.00	93.20	6.80	

**Table 4:** Gradation characteristics of coarse aggregates.

Sieve Size	Sieve Opening (mm)	Weight Retained (gm)	Cumulative Weight Retained (gm)	Cumulative Weight Retained (%)	% Finer	F. M. Value
3.0″	75.00	0.00	0.00	0.00	100.00	7.06
1.5″	37.50	0.00	0.00	0.00	100.00	
1.0″	25.40	415.00	415.00	8.30	91.70	
3/4″	19.00	572.50	987.50	19.75	80.25	
1/2″	12.50	1232.50	2220.00	44.40	55.60	
3/8″	9.50	990.00	3210.00	64.20	35.80	
#4	4.75	777.50	3987.50	79.75	20.25	
#8	2.38	560.00	4547.50	90.95	9.05	
#16	1.19	377.50	4925.00	98.50	1.50	
#30	0.59	75.00	5000.00	100.00	0.00	
#50	0.30	0.00	5000.00	100.00	0.00	
#100	0.15	0.00	5000.00	100.00	0.00	

**Table 5:** Compressive strength data, mortar mixes.

Mix ID	Compressive Stregth (MPa)				Mean Compressive Strength (MPa)	Standard Deviation (SD)	Coefficient of Variation (CV)	Standard Error (SE)	95% Confidence Interval	
	Days	S1	S2	S3					Upper Limit	Lower Limit
BLA0	7	25.28	25.60	24.95	25.28	0.325	0.013	0.188	26.084	24.469
	28	32.88	33.00	32.75	32.88	0.125	0.004	0.072	33.187	32.566
BLA5	7	25.12	24.84	24.50	24.82	0.310	0.013	0.179	25.591	24.049
	28	31.49	31.67	31.50	31.55	0.101	0.003	0.058	31.805	31.302
BLA10	7	23.65	24.00	24.15	23.93	0.257	0.011	0.148	24.571	23.296
	28	30.29	30.22	30.45	30.32	0.118	0.004	0.068	30.613	30.027
BLA15	7	22.76	23.25	23.12	23.04	0.254	0.011	0.147	23.674	22.413
	28	29.82	30.11	30.13	30.02	0.173	0.006	0.100	30.451	29.589
BLA20	7	22.55	22.67	22.95	22.72	0.205	0.009	0.119	23.233	22.213
	28	30.00	29.97	29.50	29.82	0.280	0.009	0.162	30.520	29.127
BLA25	7	21.44	21.50	21.66	21.53	0.114	0.005	0.066	21.816	21.251
	28	28.51	28.50	28.22	28.41	0.165	0.006	0.095	28.819	28.001
BLA30	7	21.00	20.85	20.55	20.80	0.229	0.011	0.132	21.369	20.231
	28	27.25	26.78	26.85	26.96	0.254	0.009	0.146	27.590	26.330

**Table 6:** Compressive strength data, concrete mixes.

Mix ID	Compressive Strength (MPa)				Mean Strength (MPa)	Standard Deviation (SD)	Coefficient of Variation (CV)	Standard Error (SE)	95% Confidence Interval	
	Days	S1	S2	S3					Upper Limit	Lower Limit
BLA0	7	28.11	28.25	28.50	28.29	0.198	0.007	0.114	28.777	27.796
	28	36.25	36.19	36.50	36.31	0.164	0.005	0.095	36.722	35.905
BLA5	7	27.71	27.51	27.73	27.65	0.122	0.004	0.070	27.952	27.348
	28	35.00	34.85	34.78	34.88	0.112	0.003	0.065	35.156	34.597
BLA10	7	26.48	26.69	26.92	26.70	0.220	0.008	0.127	27.243	26.150
	28	33.75	33.35	33.61	33.57	0.203	0.006	0.117	34.074	33.066
BLA15	7	25.71	25.58	25.85	25.71	0.135	0.005	0.078	26.049	25.378
	28	33.00	33.35	33.11	33.15	0.179	0.005	0.103	33.598	32.709
BLA20	7	25.19	25.33	25.41	25.31	0.111	0.004	0.064	25.587	25.033
	28	32.81	32.69	32.53	32.68	0.140	0.004	0.081	33.026	32.328
BLA25	7	23.57	24.00	23.75	23.77	0.216	0.009	0.125	24.310	23.237
	28	31.00	30.13	30.65	30.59	0.438	0.014	0.253	31.681	29.506
BLA30	7	22.95	22.20	22.56	22.57	0.375	0.017	0.217	23.502	21.638
	28	29.00	28.91	29.15	29.02	0.121	0.004	0.070	29.321	28.719

**Table 7:** Splitting tensile strength data, concrete mixes.

Mix ID	Splitting Tensile Strength (MPa)				Mean Strength (MPa)	Standard Deviation (SD)	Coefficient of Variation (CV)	Standard Error (SE)	95% Confidence Interval	
	Days	S1	S2	S3					Upper Limit	Lower Limit
BLA0	7	2.82	2.75	2.95	2.84	0.101	0.036	0.059	3.092	2.588
	28	4.13	4.35	3.97	4.15	0.191	0.046	0.110	4.624	3.676
BLA5	7	2.68	2.55	2.71	2.65	0.085	0.032	0.049	2.858	2.435
	28	3.86	3.99	3.62	3.82	0.188	0.049	0.108	4.290	3.357
BLA10	7	2.31	2.38	2.33	2.34	0.036	0.015	0.021	2.430	2.250
	28	3.79	3.65	4.00	3.81	0.176	0.046	0.102	4.251	3.376
BLA15	7	2.09	2.22	2.18	2.16	0.067	0.031	0.038	2.329	1.998
	28	3.67	3.40	3.76	3.61	0.187	0.052	0.108	4.075	3.145
BLA20	7	2.07	2.15	2.10	2.11	0.040	0.019	0.023	2.207	2.006
	28	3.51	3.29	3.34	3.38	0.115	0.034	0.067	3.667	3.093
BLA25	7	2.05	2.00	1.95	2.00	0.050	0.025	0.029	2.124	1.876
	28	3.19	3.25	3.31	3.25	0.060	0.018	0.035	3.399	3.101
BLA30	7	1.92	1.85	1.98	1.92	0.065	0.034	0.038	2.078	1.755
	28	3.08	3.15	3.00	3.08	0.075	0.024	0.043	3.263	2.890

Mix ID: Mix design identification; S1, S2 and S3: Sample 1, 2 and 3.

**Table 8:** Classification of pulse velocity and quality of concrete [68].

Pulse Velocity (m/s)	Concrete Quality (Grading)
Above 4500	Excellent
3500 to 4500	Good
3000 to 3500	Medium
Below 3000	Doubtful

**Table 9:** Flexural strength data for all BLA mixes.

Mix ID	Flexural Stregth (MPa)				Mean Strength (MPa)	Standard Deviation (SD)	Coefficient of Variation (CV)	Standard Error (SE)	95% Confidence Interval	
	Days	S1	S2	S3					Upper Limit	Lower Limit
BLA0	7	3.53	3.72	3.89	3.71	0.182	0.049	0.105	4.165	3.261
	28	5.00	5.43	5.38	5.27	0.236	0.045	0.136	5.854	4.683
BLA10	7	2.92	3.17	3.22	3.10	0.160	0.052	0.092	3.501	2.705
	28	4.95	5.19	5.25	5.13	0.161	0.031	0.093	5.528	4.730
BLA20	7	2.81	2.94	2.50	2.75	0.226	0.082	0.131	3.312	2.188
	28	4.73	5.21	5.08	5.01	0.246	0.049	0.142	5.617	4.394
BLA30	7	2.54	2.73	2.63	2.63	0.096	0.036	0.055	2.869	2.394
	28	4.15	3.87	4.12	4.05	0.151	0.037	0.087	4.424	3.672

Mix ID: mix design identification; S1, S2 and S3: Sample 1, 2 and 3.

## Data Availability

Data are contained within the article.
